# Supplementary figures and images for: Efficacy of Shu-yi-ning-chang decoction on IBS-D: Modulating Nr4a3 pathway to reduce visceral hypersensitivity
Source: PLoS One. 2024 Apr 17;19(4):e0299376. doi: 10.1371/journal.pone.0299376 (PMC11023393; doi:10.1371/journal.pone.0299376)

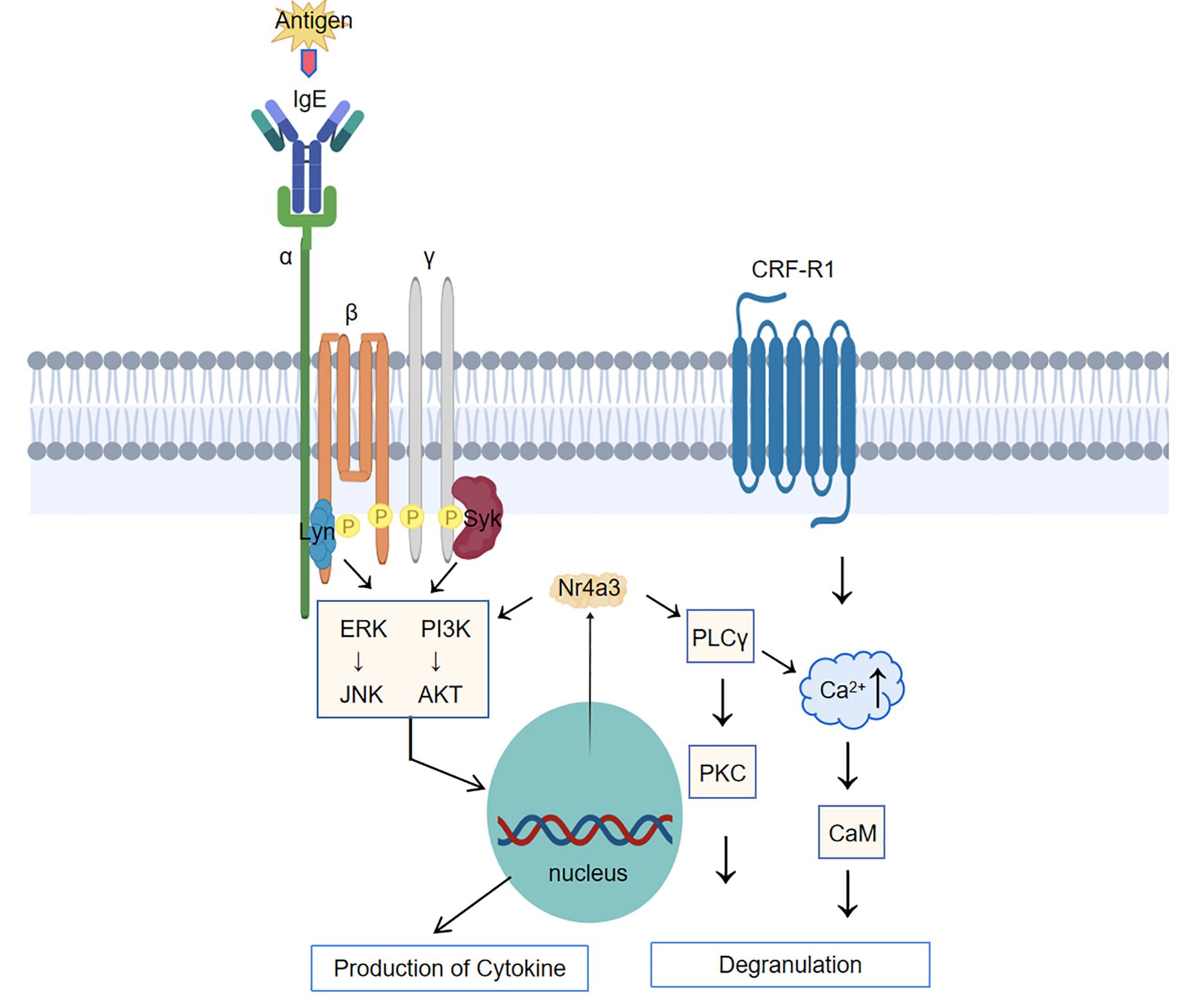

Supplement: S1 Fig — (TIF) [file pone.0299376.s001.tif]
