# Supplementary material for: Efficacy of Shu-yi-ning-chang decoction on IBS-D: Modulating Nr4a3 pathway to reduce visceral hypersensitivity
Source: PLoS One. 2024 Apr 17;19(4):e0299376. doi: 10.1371/journal.pone.0299376 (PMC11023393; doi:10.1371/journal.pone.0299376)

一. CRF-R1-1 (51KD) β-actin (42KD) Colonic tissue

CRF-R1-1

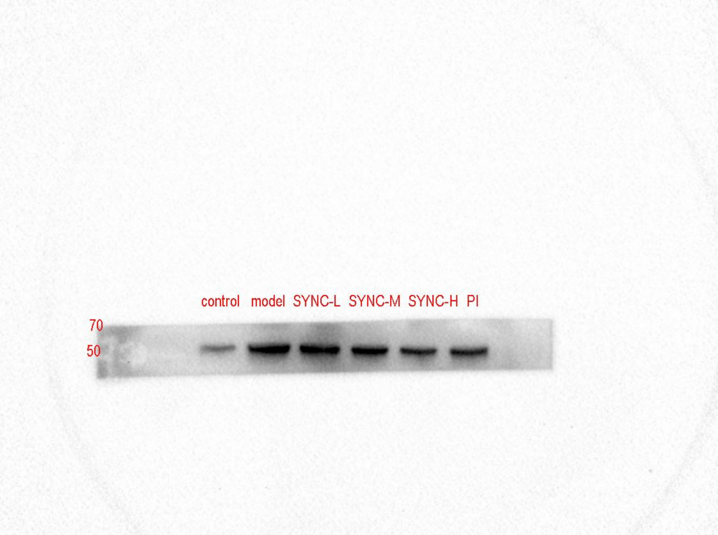

CRF-R1-2

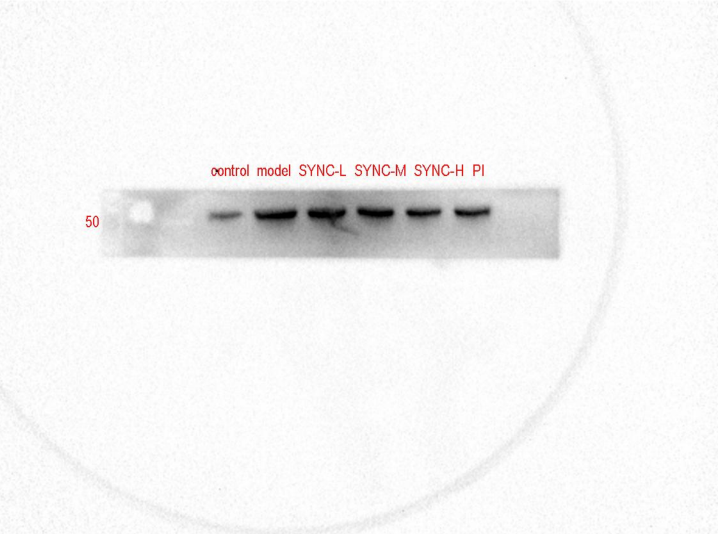

CRF-R1-3

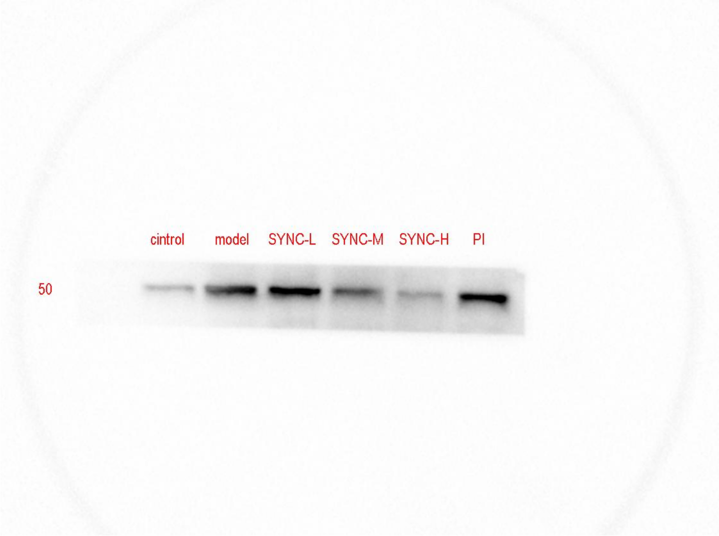

β -actin-1

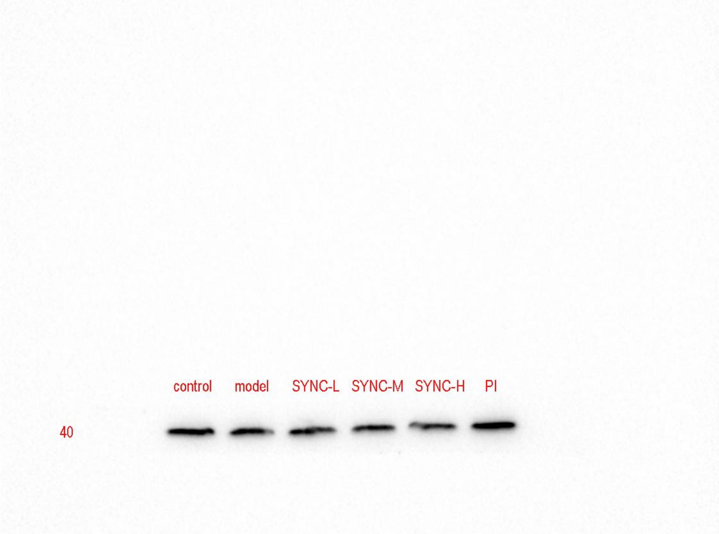

β -actin-2

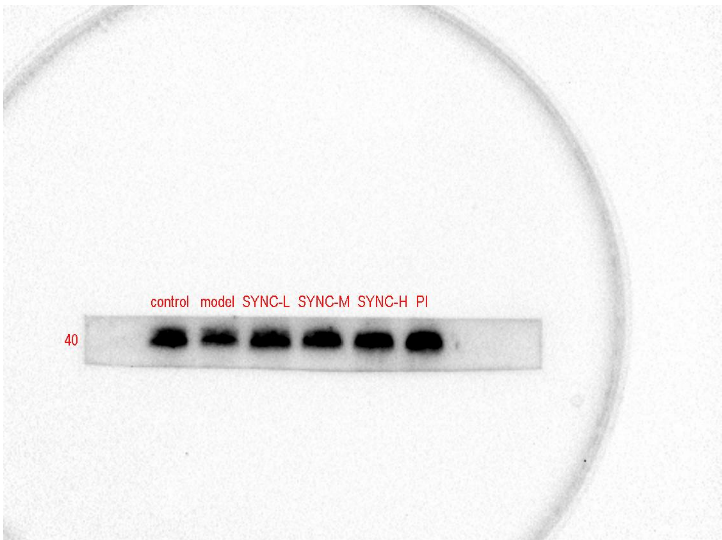

β -actin-3

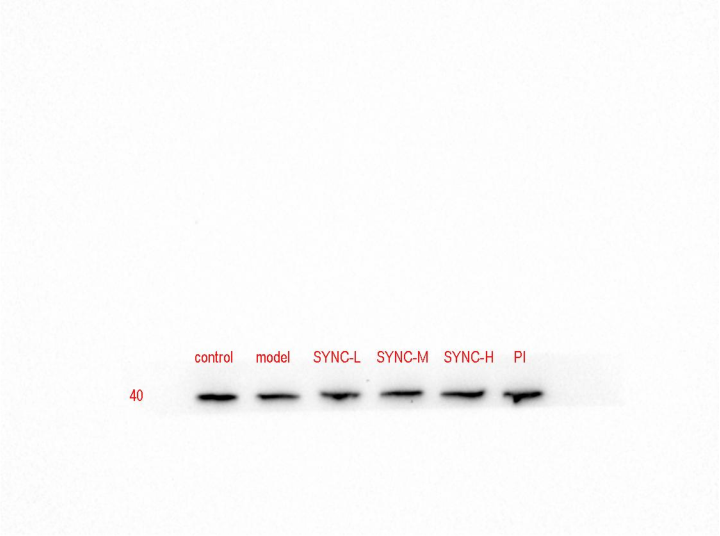

二. CRF-R1-1 (51KD) β -actin (42KD) Hypothalamic tissue

CRF-R1-1

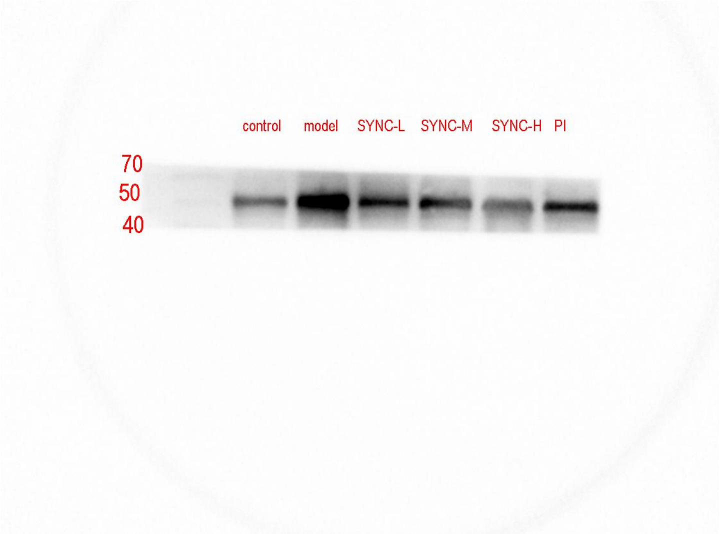

CRF-R1-2

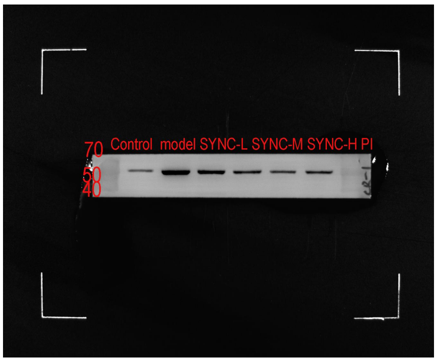

CRF-R1-3

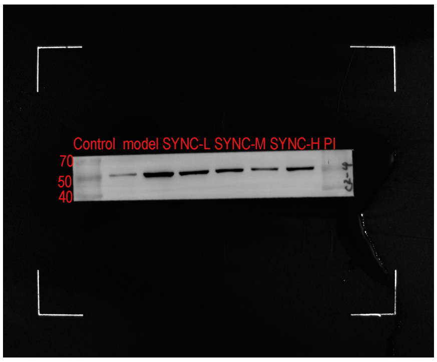

β -actin-1

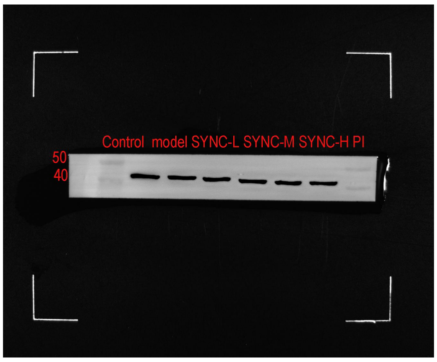

β -actin-2

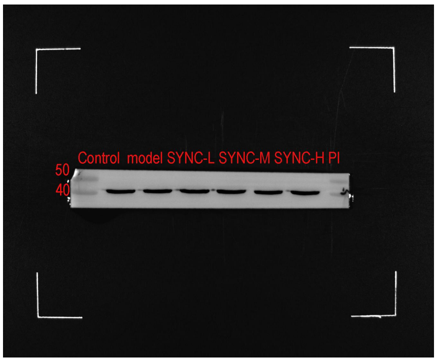

β -actin-3

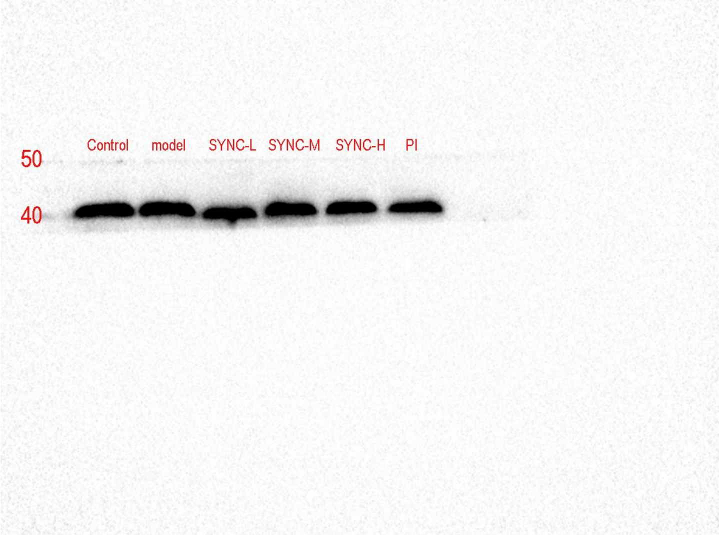

三. NR4A3 (65KD) PI3K (85KD) AKT (56KD) p-PI3K (110KD) p-AKT (56KD)
β -actin (42KD) Colonic tissue

NR4A3-1

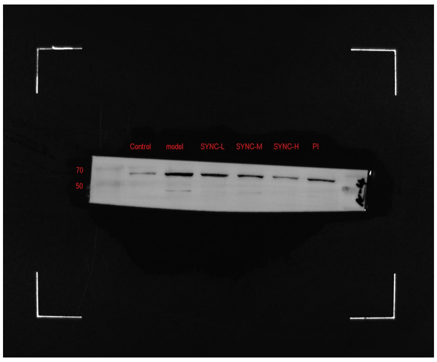

NR4A3-2

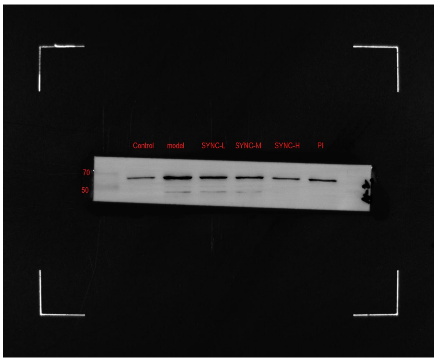

NR4A3-3

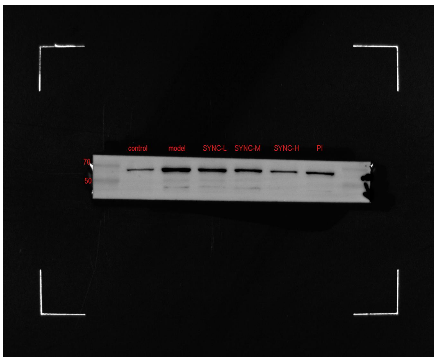

PI3K-1

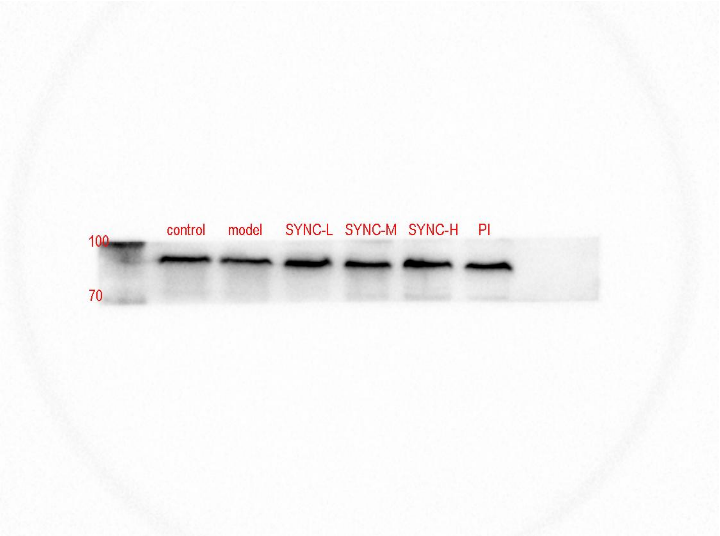

PI3K-2

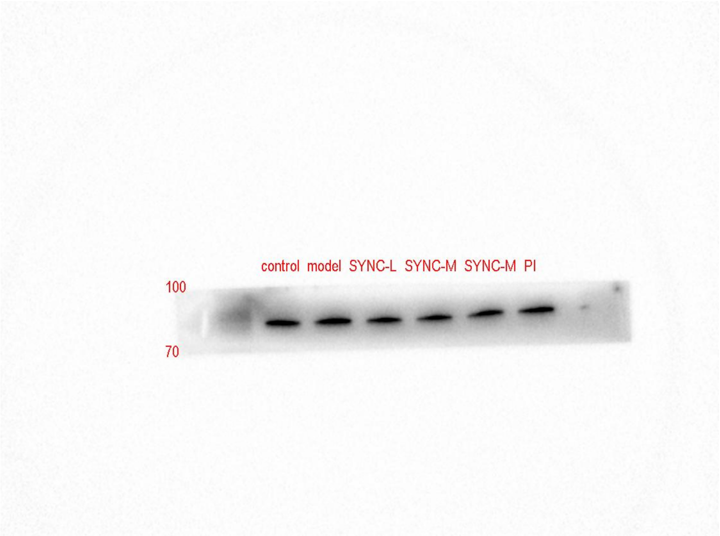

PI3K-3

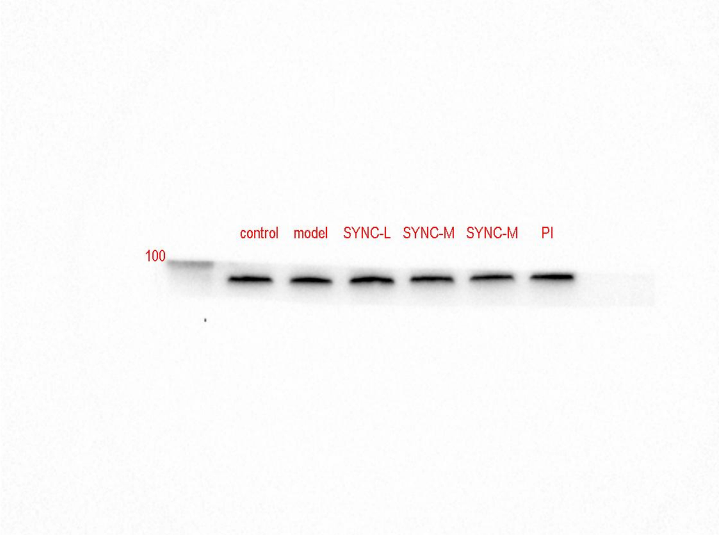

PI3K-4

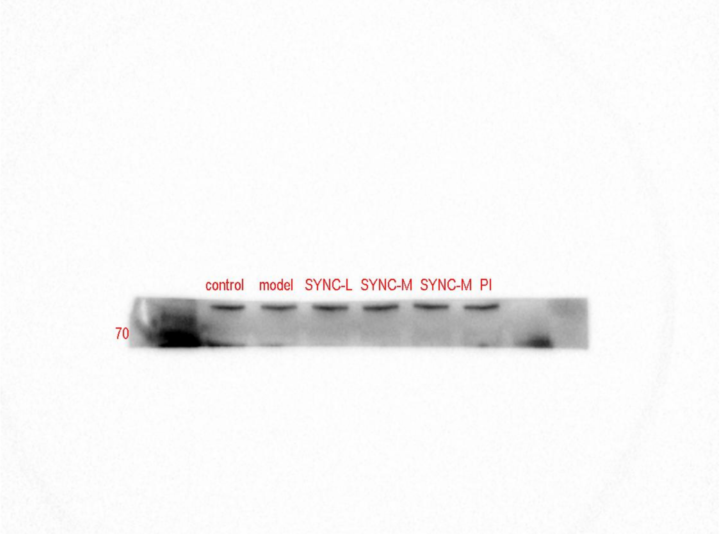

PI3K-5

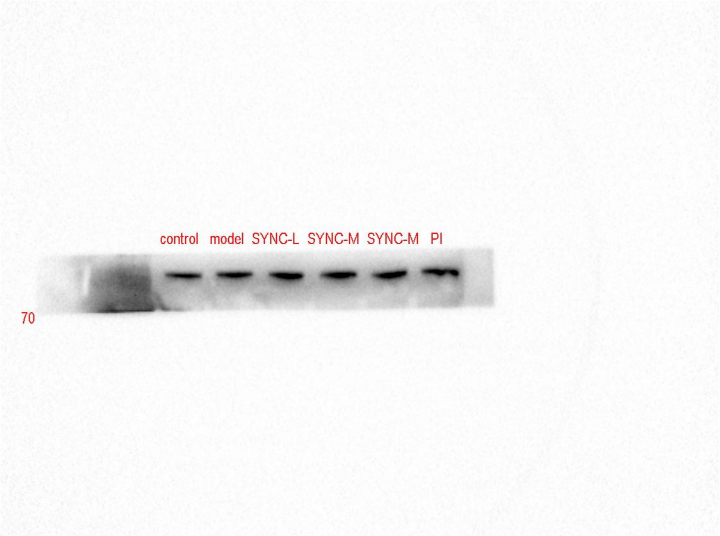

AKT-1

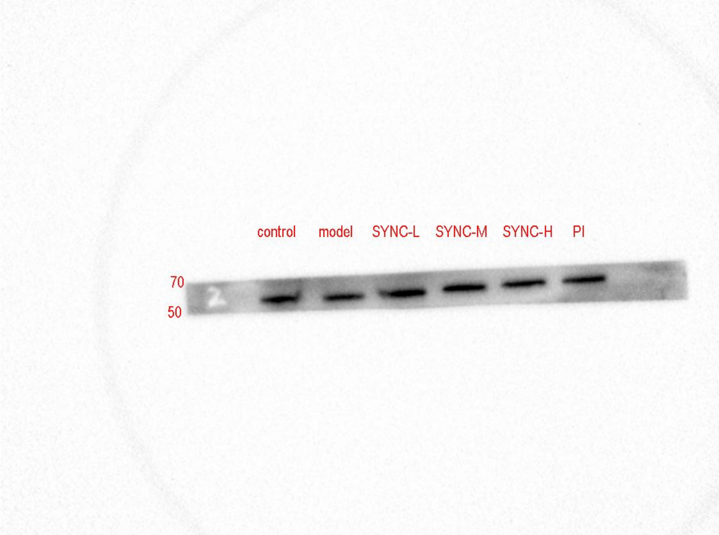

AKT-2

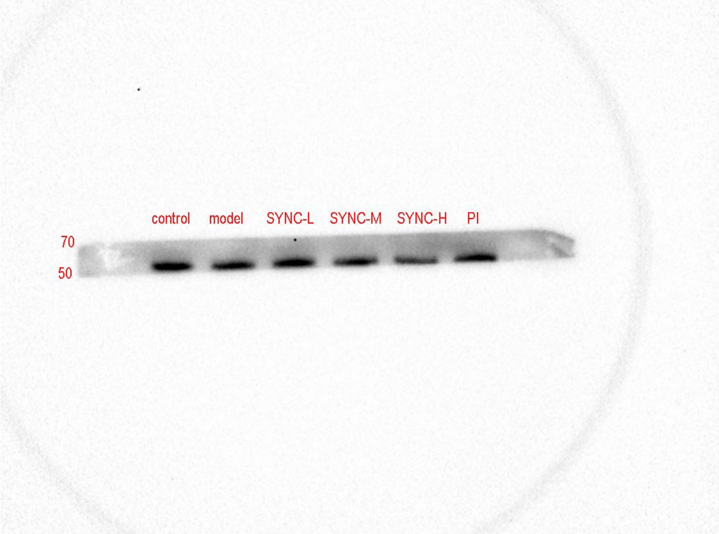

AKT-3

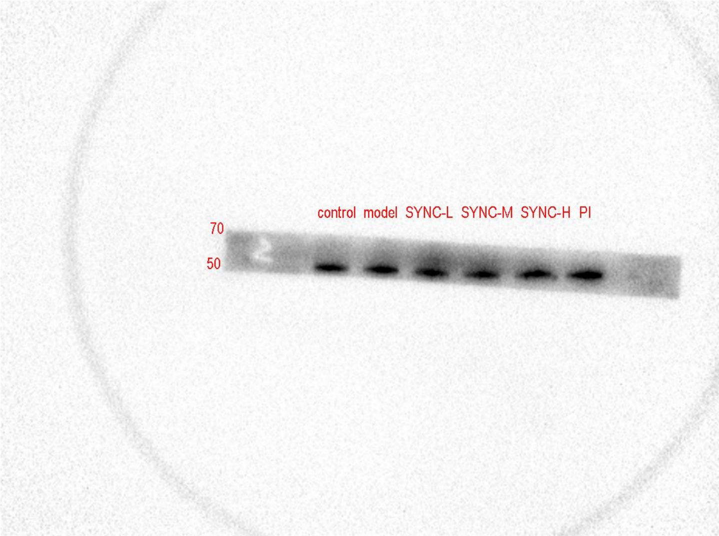

AKT-4

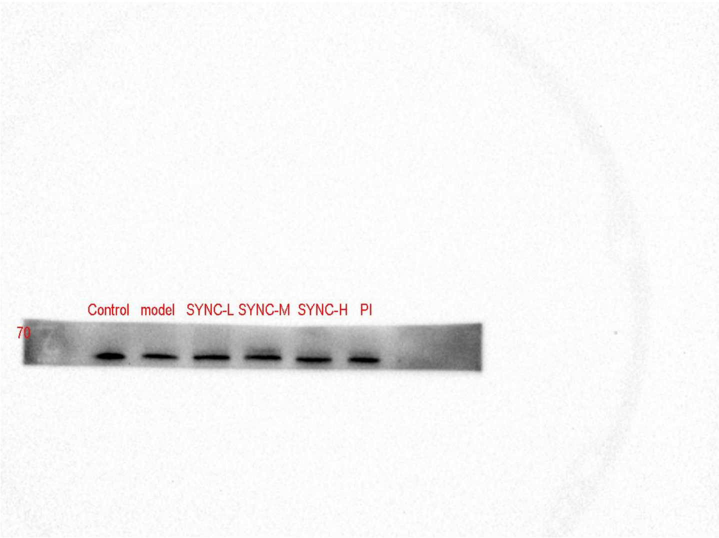

AKT-5

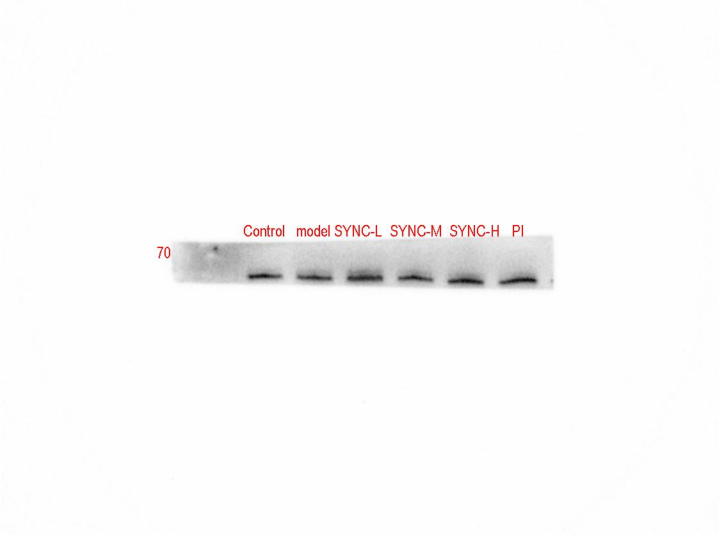

p-PI3K-1

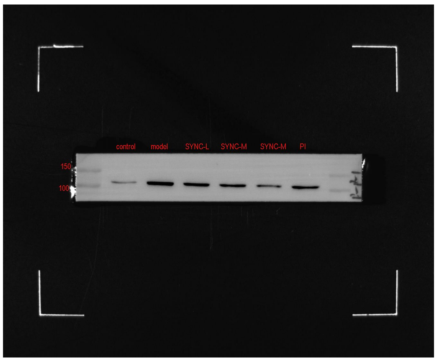

p-PI3K-2

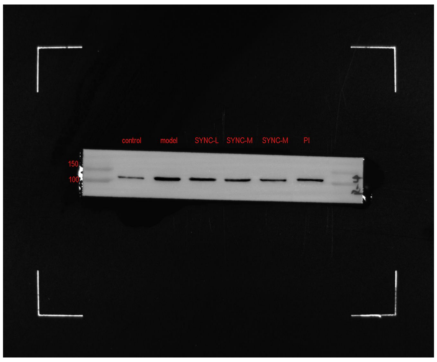

p-PI3K-3

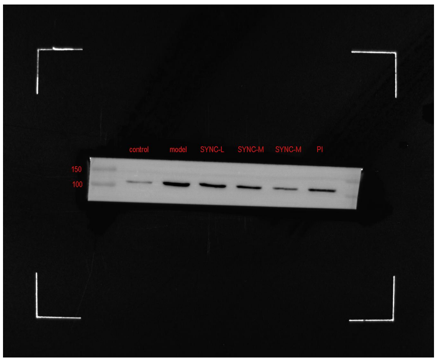

p-AKT-1

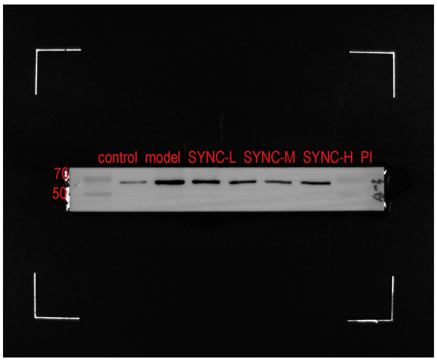

p-AKT-2

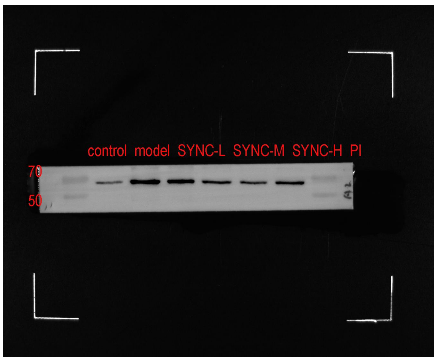

p-AKT-3

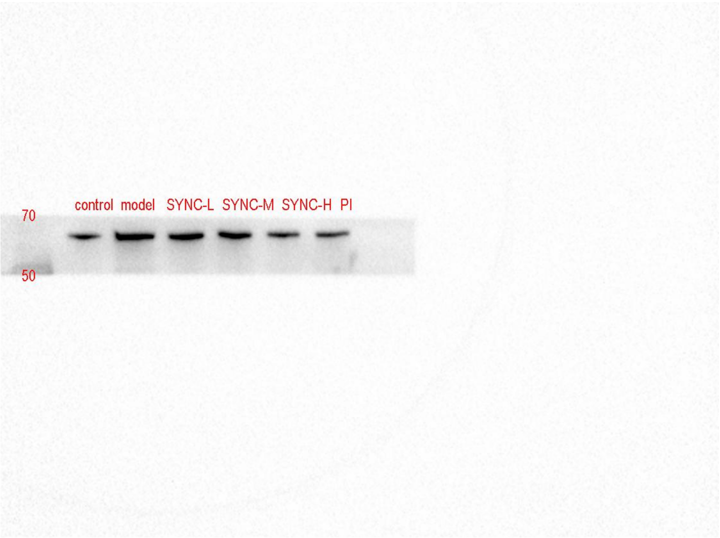

β -actin-1

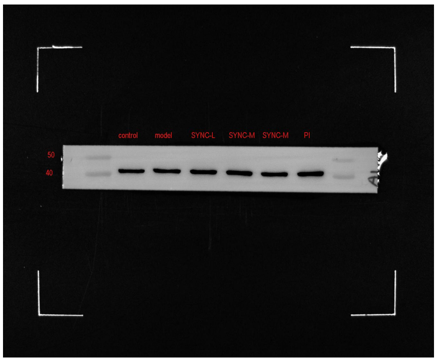

β -actin-2

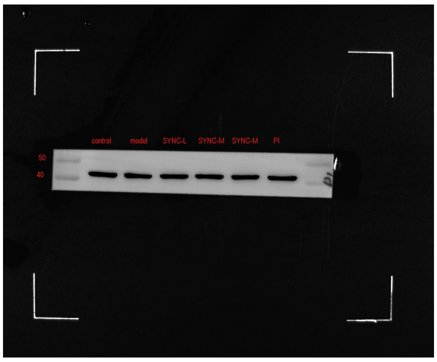

β -actin-3

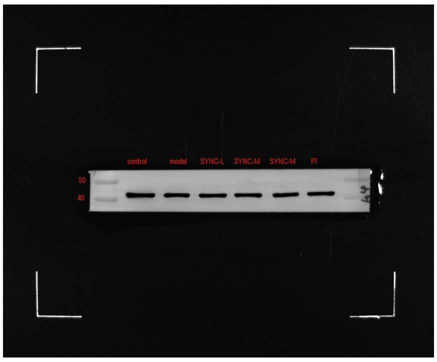

β -actin-4

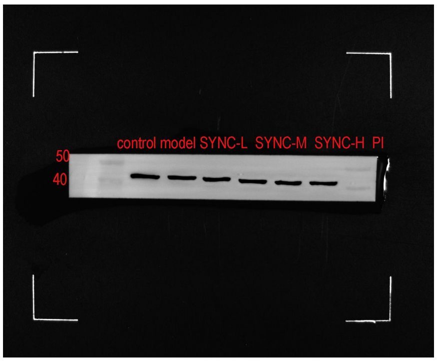

β -actin-5

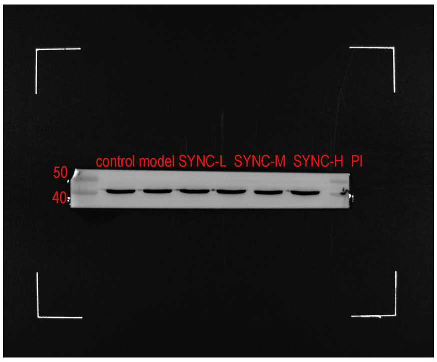

β -actin-6

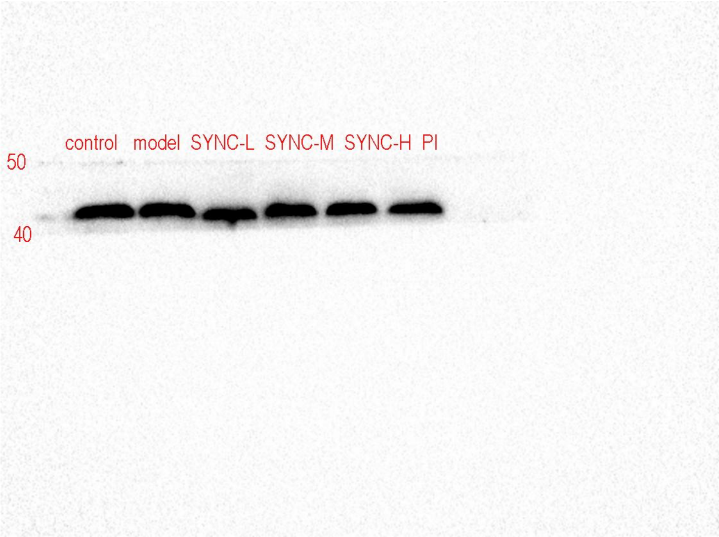

β -actin-7

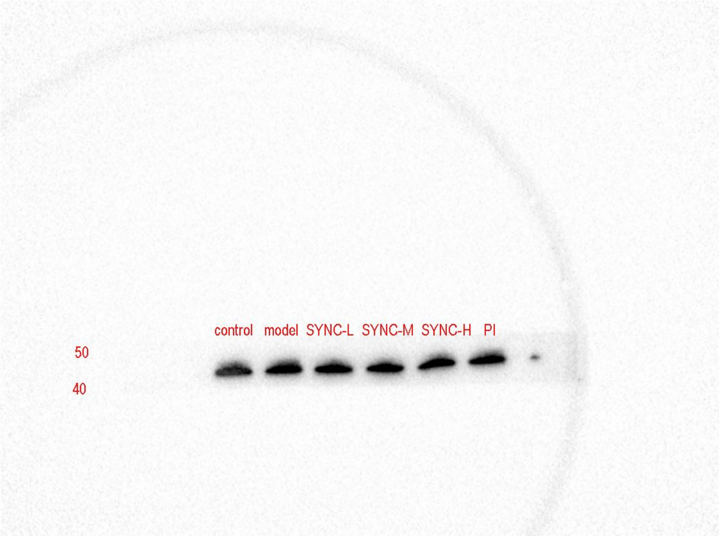

Supplement: S1 Raw images — (PDF) [file pone.0299376.s003.pdf]
