# Supplementary material for: Efficacy of Shu-yi-ning-chang decoction on IBS-D: Modulating Nr4a3 pathway to reduce visceral hypersensitivity
Source: PLoS One. 2024 Apr 17;19(4):e0299376. doi: 10.1371/journal.pone.0299376 (PMC11023393; doi:10.1371/journal.pone.0299376)
Supplement: Attachment — Submitted filename: Response to Reviewers.doc [file pone.0299376.s004.doc]

December 21, 2023

Dear Editor and Reviewers,

We gratefully thanks for the precious time the reviewers spent fmaking constructive remarks. We have carefully read the reviewers' comments and revised the manuscript accordingly, as well as provide the point-by-point responses. All changes were marked in blue using the “Track Changes” function in the revised manuscript. We have invited native English speakers to touch up the English grammar and coherence of the article (Manuscript Number: PONE-D-23-36123).

In the WB section, upon further verification, we discovered that there is no notable disparity in the PI3K and AKT expression prior to phosphorylation. So p-PI3K and p-AKT were added to make the data more complete (the images are presented in two ways due to different Gel imaging systems in the labs).

The following is a summary list of changes:

**Journal requirements**

Thank you for your advice. The revised manuscript has been ensured to meet PLOS ONE's style requirements. Animal ethics has also been added, all the data used for the drawing has been provided as Supporting Information to be uploaded into the system, ORCID information has been submitted in the website, and the original images about WB have been uploaded.

**Reviewer #1**

We appreciate your kind suggestions and we have amended the manuscript accordingly. In addition, the followings are answers for your questions:

1. Thank you for pointing out this problem in manuscript, the issue regarding the title of the article has been corrected (Efficacy of Shu-yi-ning-chang Decoction on IBS-D: Modulating Nr4a3 Pathway to Reduce Visceral Hypersensitivity).

2. We agree with this comment and rewrote this portion of the revised draft as follows: “IBS is mainly characterized by persistent or intermittent abdominal pain and bloating, which is related to changes in the frequency or form of stool. It also includes a range of physical, visceral, or psychological comorbidities”

3. As suggested by the reviewer, we have deleted "eating disorders" and cited the relevant paper (5.Ng, Q.X., Soh, A.Y.S., Loke, W., Lim, D.Y., Yeo, W.S., 2018. The role of inflammation in irritable bowel syndrome (IBS). J. Inflamm. Res. 11, 345-349. https://doi.org/10.2147/JIR.S174982).

4.As the reviewer understands, the word "palliative" does mean "symptomatic" and we have corrected it: these methods usually can only temporarily relieve symptoms, marked by limitations and high recurrence rates.

5.Based on the reviewer's question, we have rephrased "solidify the root" to make its meaning clearer: strengthening the spleen can enhance the body's resistance.

6.Thank you very much for your suggestion, we found this article very valuable and have made it a reference (49.Ng, Q.X., et al., A Meta-Analysis of the Clinical Use of Curcumin for Irritable Bowel Syndrome (IBS). J Clin Med, 2018. 7(10).)

7.Thanks to your valuable suggestions, we have added content about IBS in the introduction section on page 4.

8.Thanks to your careful review, we have modified the writing of the names of the herbs, such as *Radix bupleuri* (Chai Hu), *Angelica sinensis radix* (Dang Gui),

9.We apologize for our oversight, content on animal ethics has been added, such as the use of sodium pentobarbital (40 mg/kg, I.p) for pain relief.

10.We appreciate your feedback, we have accordingly increased the number of relevant experimental repetitions to make the experimental design clearer and more plausible.

11.We appreciate your suggestion, we have stated the exact p-values and 95% CI as much as possible in the manuscript, there is no exact p-value when p < 0.001, so it is written as p < 0.001. It is difficult to list the exact p-values for each group when there is a uniform comparison between multiple groups, so it is written as uniform p-value.

12. Thank you for pointing this out, the causal relationship between SYNC and nr4a3 does need to be further verified, we are also doing in vitro silencing and overexpression experiments of the relevant pathway using RBL-2H3 cells, and we hope to continue to collaborate with your journal. SYNC is an effective prescription for the clinical treatment of IBS-D, and clinical trials on it are ongoing. According with your advice, we have added the limitations section in the discussion section accordingly on page 25.
13. Considering the reviewer's suggestion, we address alternative pathways (IL-17 signaling pathway, NF-κB signaling pathway, TNF signaling pathway, NOD-like receptor signaling pathway and MAPK signaling pathway) related to SYNC treatment of IBS on page 24.

14.We have provided the original figures for the western blots and performed quantitative statistical analyses.

15.Following the reviewers' suggestions, we have provided a scale of Figures 3 and 5 (400×) in the revised manuscript and labeled the stains used (MCs are blue-purple after toluidine blue staining).

**Reviewer #2**

Thank you for your nice comments on our article. According to your suggestions, we have supplemented several data here and corrected several mistakes in our previous draft.

1.This section on "Identified chemical components in SYNC" is included due to the complexity of Chinese medicine compound prescriptions, which consider multiple symptoms. By utilizing HPLC, we can gain a deeper understanding of the prescription's function and gather insights for further research on its primary chemical components.

2.Thank you very much for your valuable comments. Currently, we have only analyzed the primary chemical components of SYNC based on the prescription and HPLC. This analysis was aimed at understanding the composition of the prescription. Following your suggestion, our team will focus on examining the content of SYNC in our future work.

3.We apologize for the unclear presentation and we have corrected it in the revised manuscript. There were a total of 12 pregnant rats in the animal experiments, and group experiments using offspring rats were conducted with 8 rats in each group, totaling 48 rats.

4.Thanks to your careful review, we have re-evaluated the colon pathology images to enhance the comparison between each groups and improve the clarity of the results analysis. (Compared with the normal group, the colonic mucosal structure in the model group remained largely intact, with widened intestinal glandular spaces. Numerous specimens exhibited substantial inflammatory cell infiltration and submucosal edema. In contrast to the model group, the colonic mucosa in the PI and SYNC-H groups demonstrated structural integrity, with glands arranged regularly, distinct layer structure, and a normal number of cup cells. Some inflammatory cell infiltration was observed. Relative to the SYNC-H group, the SYNC-L and SYNC-M groups exhibited more pronounced inflammatory cell infiltration in the colonic mucosa and wider gaps between intestinal glands).

5.Thank you for your suggestion, we have marked the location of the lesion in the pathological images: Black arrows points to lymphocytic infiltration, red arrows points to eosinophilic infiltration, and blue arrows indicate widening of the intestinal glandular spaces in comparison with the normal group, suggesting changes such as edema when the sample is fresh.

6.We are sorry that it is difficult to mark the exact location of the differential genes in the volcano map in Fig. 7 due to the ponderous number of genes clustered in the gray area.

7.We fully understand the concerns of the reviewers, the previous literature search revealed a connection between the Nr4a family and IBS. Specifically, Nr4a3 was found to not only influence mood through the brain-gut axis, but also to interact with mast cells and play a role in the immune-inflammatory process, which are associated with IBS. Consequently, Nr4a3 and PI3K were chosen as the primary genes of interest. We mapped the relevant pathways as follows.

8.Thank you for pointing out this problem. Based on the transcriptome results, many of the pathways impacted by SYNC are linked to immune inflammation, including the IL-17, NF-κB, TNF, NOD-like receptor and MAPK signaling pathways. This suggests that SYNC may have potential in reducing low-grade inflammation in the intestines. In the Nr4a3/PI3K pathway, which is our main focus, the Nr4a3 gene not only regulates mood but also has connections to immune inflammation. SYNC is a TCM prescription based on the holistic concept, regulating the liver qi to improves negative emotions, regulating the spleen and stomach to strengthen the gastrointestinal function, which is a comprehensive prescription.

Once again, thank you very much for your suggestions. And we hope that the revised manuscript can be accepted by *PLOS ONE*. If further revision is necessary, please contact me.

Thank you and best regards.

Sincerely yours,

Ya-Jing Guo

Corresponding author:

Name: Xiao-Jun Yang
