# Supplementary material for: Efficacy of Shu-yi-ning-chang decoction on IBS-D: Modulating Nr4a3 pathway to reduce visceral hypersensitivity
Source: PLoS One. 2024 Apr 17;19(4):e0299376. doi: 10.1371/journal.pone.0299376 (PMC11023393; doi:10.1371/journal.pone.0299376)
Supplement: Attachment — Submitted filename: Response to Reviewers.doc [file pone.0299376.s005.doc]

February 1, 2024

Dear Reviewers,

Thank you very much for your professional advice. We have carefully revised the manuscript by incorporating all the suggestions by the review panel. Once again, We have invited native English speakers to touch up the English grammar and coherence of the article (Manuscript Number: PONE-D-23-36123). At the end of the letter, we have attached the English editing certificate.

The following is a summary list of changes:

**Reviewer #1**

We appreciate your further professional review work on our article. As you are concerned, there are several problems that need to be addressed. According to your nice suggestions, we have made extensive corrections to our previous manuscript. We have added necessary sections to the manuscript to complete the article. The detailed corrections are listed below.

1. We apologize for our oversight and have reworked the grammar and phrasing of the article.

2. "... regulating the liver qi to improves negative emotions, regulating the spleen and stomach to strengthen the gastrointestinal function, which is a comprehensive prescription" is a TCM term in Chinese medicine theory, meaning that regulating the qi of the liver and the function of the spleen and stomach can achieve the effect of improving depression and strengthening the function of the intestinal tract, and this method is extremely effective in treating IBS.

3. Thank you very much for your very useful suggestions, as we have elaborated on the specific dosages, methods of administration and evaluation criteria in the manuscript. The dosage of each Chinese medicine is supplemented in detail in the drug component. The section "Identified chemical components in SYNC" has been deleted at the suggestion of reviewer 2.

**Specifics on dosages:** Radix bupleuri (Chai Hu) 15g, Angelica sinensis radix (Dang Gui) 10g, Fructus Aurantii (Zhi Qiao) 10g, Radix Paeoniae Alba (Bai Shao) 10g, Rhizoma Corydalis(Yuan Hu) 10g, Radix Pseudostellariae (Tai Zi Shen) 15g, Poria (Fu Ling) 10g, Rhizoma Atractylodis Macrocephalae (Bai Zhu) 10g, Dried tangerine peel (Chen Pi) 10g, Pericarpium Granati (Shi Liu Pi) 10g, Bergamot (Fo Shou) 10g, Citron (Xiang Yuan) 10g.

**Dosages and methods of administration:** Successfully modeled rats were randomly assigned to one of five groups, the model group, SYNC low-dose (6.825 g/kg/d), medium-dose (13.65 g/kg/d), high-dose (27.3 g/kg/d), and Pinaverium Bromide group (15.75 mg/kg/d). The drug dosages were adjusted based on equivalent dosages for adults and rats and administered via gavage for 14 days.

**Criteria for evaluation:** **1. Fecal water content.** Fecal water content (%) was determined by collecting feces from each rat before and after molding between 8:00 and 9:00. The collected feces were weighed and recorded as wet weight. Next, they were dried in an oven at 120℃ for 30 min and weighed again, with this recorded as the dry weight. This process was repeated until the weight of the feces no longer changed. Fecal water content was calculated as follows: feces water content (%) = (wet weight of feces+dry weight of feces)/ wet weight of feces×100% [13]). **2.** **Body weight:**Additionally, rat body weights were recorded every five days, starting from the end of the modeling period (week 0) and continuing until the conclusion of the experiment, totaling six measurements. **3. Abdominal withdrawal reflex (AWR) scoring:** AWR scores were assigned to rats before and after treatment. Rats were fasted but provided with water 24 h before measurement. A catheter lubricated with paraffin oil was gently inserted into the rat’s anus and secured to the tail with medical tape to prevent dislodgment. The rats were then placed in a transparent container, and room-temperature pure water was injected into a balloon through the catheter. Each injection lasted for 20 s, and the water volume injected was noted when the AWR score was 3. This process was repeated 3 times with a 15 min interval between injections, and the average value was used to determine the minimum volume threshold for AWR caused by non-injurious colorectal dilatation in these rats.

4. Thanks to your valuable suggestions, we added a section on drug safety to the discussion of the manuscript.

(Safety is the most important consideration during the design of pharmaceuticals. The complex and specific nature of traditional Chinese medicine has raised concerns about its safety. In response, China established an adverse drug reaction monitoring system in 1999. According to the *Annual Report on National Adverse Drug Reaction Detection*, Chinese medicine, excluding injections, generally exhibit favorable safety. In our study, the SYNC formula was orally administered, which maximizes its safety. None of the drugs in SYNC fall under categories with toxic side effects, such as "Eighteen incompatible herbs and Nineteen herbs of mutual antagonism." Furthermore, SYNC did not show any notable toxic side effects in clinical practice. However, these observations do not imply absolute safety, and we aim to explore its long-term safety to prevent potential toxic side effects. Moreover, considering the personalized nature of TCM prescriptions, clinical treatment should be tailored according to individual symptoms).

**Reviewer #2**

We appreciate your detailed and constructive comments. According to your suggestions, we have supplemented relevant content here and corrected several mistakes in our previous manuscript. We deleted some unneeded content and explained and revised the manuscript according to each suggestion one by one. The detailed corrections are listed below.

1. Thank you very much for your suggestion. After careful consideration, we have deleted the section "Identified chemical components in SYNC".

2. We apologize for our oversight and have carefully revised the formatting of the manuscript to ensure compliance with *PLOS ONE*.

3. Thanks to your proposal, we have mapped the relevant access roads as follows:

(To prevent the image from being lost again, name the image in the article submission system: Signaling Pathway)

4. We appreciate your valuable feedback, and we have included an analysis of the pathways and genes impacted by SYNC in the manuscript：

(KEGG and GO enrichment analysis also identified additional pathways potentially associated with SYNC's treatment of IBS, such as the IL-17 signaling pathway, NF-κB signaling pathway, TNF signaling pathway, NOD-like receptor signaling pathway, and MAPK signaling pathway, all of which are associated with the alleviation of low-grade inflammation and reduction of symptoms associated with IBS by SYNC. In addition, NF-κB and MAPK signaling pathways are involved in the regulation of depression. Cytokine stress can induce depression and also promote inflammatory responses by regulating sympathetic and parasympathetic nervous system pathways. Depressive symptoms may arise as a behavioral consequence of the evolutionary advantage provided by genes promoting inflammation. Targeting pro-inflammatory cytokines and their signaling pathways presents a novel approach to treating depression. Building on animal experiments, we propose that SYNC can alleviate depression by downregulating the expression of CRF-R1 and Nr4a3 in the hypothalamus. In addition, it may reduce visceral hypersensitivity by inhibiting mast cell activation through the decreased phosphorylation of Nr4a3 and PI3K).

Once again, thank you very much for your suggestions. And we hope that the revised manuscript can be accepted by *PLOS ONE*. If further revision is necessary, please contact me.

Thank you and best regards.

Sincerely yours,

Ya-Jing Guo

Corresponding author:

Name: Xiao-Jun Yang
